# Factors associated with the use of supplemental oxygen or positive pressure ventilation in the delivery room, in infants born with a gestational age ≥ 34 weeks

**DOI:** 10.1186/s12978-016-0235-8

**Published:** 2016-10-17

**Authors:** Maria Elisabeth Moreira, Ana Paula Esteves Pereira, Saint Clair Gomes Junior, Ruth Guinsburg, Maria Fernanda Branco de Almeida, Silvana Granado Gama, Maria do Carmo Leal

**Affiliations:** 1 Fundação Oswaldo Cruz/Instituto Fernandes Figueira, Avenida Rui Barbosa 716, Rio de Janeiro, RJ 22520-020 Brazil; 2 Fundação Oswaldo Cruz/Escola Nacional de Saúde Publica, R. Leopoldo Bulhões, 1480 - Benfica, Rio de Janeiro, RJ 20911-300 Brazil; 3 Universidade Federal de São Paulo, São Paulo, SP Brazil

**Keywords:** C-section, Late preterm, Early term, Positive pressure ventilation

## Abstract

**Background:**

Approximately 5–10 % of newborns require some form of resuscitationupon delivery; several factors, such as maternal abnormal conditions, gestational age and type of delivery could be responsible for this trend. This study aimed to describe the factors associated with the need for positive pressure ventilation (PPV) via a mask or endotracheal tube and the use of supplemental O2 in newborns with a gestational age greater than 34 weeks in Brazil.

**Methods:**

We performed a cross-sectional study and obtained data from the Birth in Brazil Survey. The inclusion criterion was a gestational age ≥34 weeks. Exclusion criteria were newborns with congenital malformations, and cases with undetermined gestational age or type of delivery (vaginal, pre labor cesarean section and cesarean section during labor). The primary outcomes were need of PPV via a mask or endotracheal tube and the use of supplemental oxygen without PPV. Confounding variables, including maternal age, source of birth payment, years of maternal schooling, previous birth, newborn presentation, multiple pregnancy, and maternal obstetric risk, were analyzed.

**Results:**

We included 22,720 newborns. Of these, 2974 (13.1 %) required supplementary oxygen. PPV with a bag and mask was used for 727 (3.2 %) newborns and tracheal intubation for 192 (0.8 %) newborns. Chest compression was necessary for 136 (0.6 %) newborns and drugs administered in 114 (0.5 %). 51.3 % of newborns were delivered by cesarean section, with the majority of cesarean sections (88.7 %) being performed prior to labor. Gestational age (late preterm infants: (Relative Risk-(RR) 2.46; 95 % (Confidence interval-CI 1.79–3.39), maternal obstetric risk (RR 1.59; 95 % CI1.30–1.94), and maternal age of 12–19 years old (RR 1.36; 95 % CI1.06–1.74) contributed to rates of PPV in the logistic regression analysis. Newborns aged between 37–38 weeks of gestaional age weren´t less likely to require PPV compared with those aged 39–41 weeks of gestational age.

**Conclusions:**

Late preterm infants, previous maternal obstetric risks and maternal age contributed to the higher needs of PPV and use of O2 in the delivery room. These variables need to be considered in planning care in the delivery room.

## Background

Approximately 5–10 % of newborns require some form of resuscitation, ranging from simple maneuvers, to assisted ventilatorysupport [1]. Neonatal Resuscitation Algorithms was recently reviewed but, since the previous update in 2010 [2], the international clinical guidelines describe an approach to newborn resuscitation which recommends the avoidance of 100 % oxygen in order to minimize the oxidative stress. Effective positive pressure ventilation (PPV) remains the key to successful resuscitation in neonates who fail to establish spontaneous breathing [3]. However, there are substantial differences in neonatal resuscitation practices in different neonatal centers, which accounts for the varying rates of PPV (positive pressure ventilation) [4].

Perinatal Risk assessment is essential to be prepared for neonatal resuscitation. The rate of resuscitation is higher if newborns are born by cesarean section (C-section) or if they are preterm infants [5]. Late preterm infants are more likely to require resuscitative procedures in the delivery room, most likely resulting from the immaturity of physiological systems in responding to stress factors during labor and birth [6, 7].

We are hypothesizing that maternal demographic characteristics, along with certain obstetric practices, could affect the necessity of PPV in the delivery room. This study aimed to describe the factors associated with the need for positive pressure ventilation (PPV) via a mask or endotracheal tube and the use of supplementary O2 in newborns with a gestational age of more than 34 weeks in Brazil.

## Methods

The “Birth in Brazil study” is a national hospital-based study of postpartum women and their newborns, carried out from February 2011 to October 2012 [8]. This study included a total of 23,894 subjects and 24,061 live births. Data weighting was calculated according to the inverse of the probability of inclusion of each puerperal woman in the sample. To ensure that the distribution of the puerperal women who were interviewed was similar to that observed among the births in the population sampled in 2011, a calibration procedure was used in each selected stratum [9]. Data on women and newborns were collected from their medical records, and photographs of the prenatal cards were taken.

This study included newborn infants ≥ 34 weeks of gestational age. Exclusion criteria included those with an undetermined gestational age (7), GA < 34 weeks (784), undetermined type of delivery (482) and newborns with severe congenital malformations in the maternal or the newborn chart (68). After applying inclusion and exclusion criteria, the final sample was 22,720 newborns. All analyses took into consideration the design of the sample and the results presented were adjusted according to the sample weight.

Post-hoc calculations showed that with a significance level of 5 %, and considering a 10 % prevalence of newborns who received an intervention during birth, the effectiveness of the sample is to detect differences of at least 2 % is 90 %.

Two groups of outcome variables of care provided to newborns were considered: the need for positive pressure ventilation (PPV) via a mask or endotracheal tube and oxygen delivered through a face mask or a hand cupped around oxygen tubing in the delivery room (supplementary oxygen). Other approaches to resuscitation are also described. We used the following independent variables to assess the varying need for resuscitation procedures in the delivery room: maternal age (<20, 20–34, and ≥ 35 years), schooling (incomplete primary education, complete primary education, complete secondary education, and complete higher education), type of delivery (vaginal, C-section in labor meaning emergency c-section, and pre-labor C-section meaning elective c-section), and source of payment for childbirth (public or private) as birth-care characteristics. We also analyzed previous birth (0, 1–2, ≥ 3), newborn presentation (cephalic, non-cephalic), type of pregnancy (single or multiple), obstetric risk (hypertensive disorders, diabetes, abruption placenta, placental previa, and intrauterine growth restriction) and the composite of all the maternal disorders. All of the selected outcome and independent variables were collected from information that was recorded on hospital charts. Information was gathered via the use of a questionnaire completed by the women.

We analyzed the differences in sociodemographic characteristics according to type of delivery by χ^2^ test. Factors associated with the use of PPV and supplementaryoxygenwere examined using univariate and multivariate logistic regression. All independent variables were included in the multivariate model. The adjusted odds ratio (OR) and respective 95%CI were estimated for all exposure variables. Interactions between the investigated variables were tested. All of the analyses were performed considering a significance level of 0.05.

The study was approved by the Ethics Research Committee of the Oswaldo Cruz Foundation, and by the ethics committees of the participating institutions. All postpartum women who were subjects in this study signed an informed consent form.

## Results

Of the 22,720 newborns included in our analysis, 2,974 (13.1 %) required supplementary oxygen in the delivery room. Ventilation with a bag and mask was necessaryfor727 (3.2 %) newborns and tracheal intubation for 192 (0.8 %) newborns. Chest compression was necessary for 136 (0.6 %) newborns and drugs were administered in 114 (0.5 %) newborns.

Delivery by C-section was performed in 51.3 % of newborns with the majority (88.7 %) performed prior to labor. Maternal demographic characteristics of the study group are shown in Table 1.

**Table 1:** Sociodemographic characteristics and type of delivery in newborns with ≥ 34 weeks/gestation

	Vaginal (*n* = 11,074)	Prelabor C-section (*n* = 10,331)	Intrapartum C-section (*n* = 1,315)	*P*-value*
	n (%)	n (%)	n (%)	
Source of payment of birth				
Public	10,512 (94.9)	6,368 (61.6)	1,140 (86.7)	<0,001
Private	562 (5.1)	3,963 (38.4)	175 (13.3)	
Age in years (11,071; 10,328; 1,315)				
12–19	2,739 (24.7)	1,218 (11.8)	312 (23.7)	<0,001
20–34	7,505 (67.8)	7,664 (74.2)	892 (67.8)	
≥ 35	827 (7.5)	1,446 (14.0)	111 (8.4)	
Years of schooling (11,030; 10,267; 1,310)				
≤ 7	3,815 (34.6)	1,791 (17.4)	373 (28.5)	<0,001
8–10	3,303 (29.9)	2,155 (21)	320 (24.4)	
11–14	3,617 (32.8)	4,650 (45.3)	533 (40.7)	
≥ 15	295 (2.7)	1,671 (16.3)	84 (6.4)	
Previous births				
0	4,696 (42.6)	5,126 (46.5)	717 (6.5)	<0,001
1–2	4,844 (43.9)	4,501 (40.8)	481 (4.4)	
≥ 3	1,534 (13.9)	704 (6.4)	117 (1.1)	
Fetal presentation				
Cefalic	11,022 (99.5)	9,599 (92.9)	1,177 (89.5)	<0,001
Non-cefalic	52 (0.5)	732 (7.1)	138 (10.5)	
Type of pregnancy				
Single	11,007 (99.4)	9,994 (96.7)	1,256 (95.5)	<0,001
Multiple	67 (0.6)	337 (3.3)	59 (4.5)	
Obstetric risk				
Any	1,410 (12.7)	3,283 (31.8)	377 (28.7)	<0,001
Hypertensive disorders	601 (5.4)	1,680 (16.3)	136 (10.3)	<0,001
Diabetes (pre-gestational or gestational)	799 (7.2)	1,047 (10.1)	105 (8.0)	<0,001
Abruptio placentae	33 (0.3)	154 (1.5)	31 (2.4)	<0,001
Placental praevia	17 (0.2)	76 (0.7)	7 (0.5)	<0,001
Intra uterine growth restriction	55 (0.5)	902 (8.7)	160 (12.2)	<0,001
Gestational age in weeks				
34–36	860 (7.8)	991 (9.6)	151 (11.5)	<0,001
37–38	3,671 (33.1)	4,189 (40.5)	416 (31.6)	
39–41	6,237 (56.3)	4,915 (47.6)	713 (54.2)	
42 or more	306 (2.8)	236 (2.3)	35 (2.7)	

* χ2 tests

PPV via mask or endotracheal tube was more frequent in the public setting, while the use of supplementary oxygen was more frequent in the private setting. Obstetric risk, except for placenta praevia, contributed to the increased need for PPV and supplementary oxygen in the delivery room (Table 2).

**Table 2:** Sociodemographic characteristics associated with PPV and supplemental O2 in the delivery room in newborns with ≥ 34 weeks/gestation

	PPV		*P*-value	Supplemental O2		*P*-value*
	n	%		n	%	
Source of payment of birth						
Public (18,021)	755	4.2	0.029	2,272	12.6	<0.001
Private (4,700)	164	3.5		701	14.9	
Age in years						
12–19 (4,269)	210	4.9	<0.001	519	12.2	0.013
20–34 (16,061)	591	3.7		2,104	13.1	
≥ 35 (2,384)	116	4.9		350	14.7	
Years of schooling						
≤ 7 (5,979)	225	3.8	0.494	686	11.5	<0.001
8 to 10 (5,778)	236	4.1		727	12.6	
11 to 14 (8,800)	370	4.2		1,243	14.1	
≥ 15 (2,050)	76	3.7		303	14.8	
Previous births						
0 (10,539)	480	4.6	0.001	1,488	14.1	<0.001
1–2 (9,826)	356	3.6		1,230	12.5	
≥ 3 (2,355)	83	3.5		255	10.8	
Fetal presentation						
Cefalic (21,798)	877	4.0	0.407	2,815	12.9	<0.001
Non-cefalic (*n* = 922)	42	4.6		159	17.3	
Type of pregnancy						
Single (22,257)	883	4.0	<0.001	2,857	12.8	<0.001
Multiple (463)	36	7.8		117	25.3	
Obstetric risk						
Any (5,072)	303	6.0	<0.001	883	17.4	<0.001
Hypertensive disorders (2,417)	151	6.3	<0.001	443	18.4	<0.001
Diabetes (pre-gestational or gestational) (1,951)	105	5.4	0.002	318	16.3	<0.001
Abruptio placentae (219)	26	12.0	<0.001	50	23.0	<0.001
Placental praevia (100)	6	6.0	0.322	24	23.8	0.001
Intra uterine growth restriction (1,118)	83	7.5	<0.001	211	18.9	<0.001
Gestational age in weeks						
34–36 (2,002)	180	9.0	<0.001	469	23.5	<0.001
37–38 (8,276)	274	3.3		990	12.0	
39–41 (11,865)	430	3.6		1,441	12.2	
42 or more (577)	36	6.3		74	12.8	
Type of delivery						
Vaginal (11,074)	419	3.8	0.002	1,137	10.3	<0.001
Prelabor C-section (10,331)	424	4.1		1,610	15.6	
Intrapartum C-section (1,315)	76	5.8		227	17.3	
Total: 22,720	919	4.0		2,974	13.1	

* χ2 tests

In the logistic regression analysis adjusted by a composite of obstetric risk, multiple or single pregnancy, years of schooling and gestational age, only the extremes of maternal age, the combined obstetric risk and gestational age increased the need for PPV (Table 3).

**Table 3:** Logistic regression analysis for PPV and supplemental O2 in the delivery room in newborns with ≥ 34 weeks/gestation

	PPV		Supplemental O2	
	OR adj.	95 % IC	OR adj.	95 % IC
Source of payment of birth				
Public	1.00	-	1.00	-
Private	0.78	(0.56 - 1.07)	0.96	(0.73 – 1.26)
Age in years				
12–19	1.36	(1.06 - 1.74)	0.94	(0.77 – 1.15)
20–34	1.00	-	1.00	-
≥ 35	1.32	(1.02 - 1.74)	1.09	(0.91 – 1.32)
Years of schooling				
≤ 7	0.91	(0.57 - 1.44)	0.96	(0.66 – 1.39)
8–10	0.95	(0.60 - 1.49)	1.01	(0.72 – 1.41)
11–14	1.06	(0.68 - 1.64)	1.06	(0.80 – 1.4)
≥ 15	1.00	-	1.00	-
Previous births				
0	1.12	(0.85 - 1.48)	1.13	(0.96 – 1.32)
1–2	1.00	-	1.00	-
≥ 3	0.86	(0.60 - 1.22)	0.88	(0.71 – 1.09)
Fetal presentation				
Cefalic	1.00	-	1.00	-
Non-cefalic	0.95	(0.63 - 1.44)	1.08	(0.86 – 1.37)
Type of pregnancy				
Single	1.00	-	1.00	-
Multiple	1.22	(0.47 - 3.13)	1.36	(0.76 – 2.41)
Any obstetric risk	1.59	(1.30 - 1.94)	1.34	(1.17 – 1.53)
Gestational age in weeks				
34–36	2.46	(1.79 - 3.39)	1.96	(1.57 – 2.45)
37–38	0.90	(0.74 - 1.10)	0.94	(0.83 – 1.06)
39–41	1.00	-	1.00	-
42 or more	1.85	(0.55 - 6.25)	1.08	(0.56 – 2.1)
Type of delivery				
Vaginal (11,075)	1.00	-	1.00	-
Prelabor C-section (10,331)	1.04	(0.79 - 1.39)	0.69	(0.56 – 0.84)
Intrapartum C-section (1,314)	1.32	(0.95 - 1.84)	1.11	(0.88 – 1.41)

## Discussion

The present study showed that late preterm infants needed more PPV in the delivery room compared with other gestational ages. As expected, late preterm neonates required more resuscitation procedures during their transition from the intra- to extra-uterine environment. According to Escobar et al. [10], greater attention should be given to clinical management, intervention, and follow-up of late preterm newborns, with the need for structured research in this area. The present study analyzed aspects that have not been extensively covered by investigators, such as the need for resuscitation procedures in the delivery room. However, importantly, PPV was not associated with delivery by C-section as shown by logistic regression analysis.

Late preterm and early term are associated with increased morbidity including the need for resuscitation in the delivery room. A cohort study in the United States on elective C-sections showed that more than one third of deliveries were performed before 39 complete weeks of gestation [11]. Additionally, the children born were at a higher risk of mortality and several other adverse neonatal occurrences, including the need for cardio-pulmonary resuscitation in the first 24 h of life [11].

An interesting finding in our study was that neonates who were born between 37 and 38 weeks of gestational age were not more likely to need PPV compared with those born between 39 and 41 weeks of gestational age. Three recent observational studies, which did not study morbidity in the delivery room, consolidated previous findings of an increased risk of neonatal composite morbidity, respiratory morbidity, and neonatal admission with elective cesarean delivery at 38 weeks of gestation compared with 39 weeks of gestation [11–13]. In contrast, results from the first randomized trial were recently reported in which there was no significant difference in the risk of neonatal admission with elective cesarean delivery between these two gestational weeks [14]. Our results are similar to those of De Almeida et al. [5]. They did not find any differences in the need for PPV provided by mask or endotracheal tube between 37–38 and 39–41 weeks of gestational age. Unfortunately, one of the limitations of this study was not having followed these newborn, so we have no information about the follow-up of these babies.

With regard to use of supplementary oxygen in the delivery room, we observed that 13.1 % of newborns had received supplementary oxygen, not in the resuscitation sequence, but as a first maneuver. This oxygen was probably used inappropriately through a face mask. Since 2010, the use of oxygen in healthy newborns in the delivery room has been considered as not necessary [2] but this is still performed in delivery rooms in Brazil. The same result was found in a previous analysis using only term newborns without risks [15].

At all gestational ages, the risks of prolonging pregnancy must be carefully weighed up against the adverse risks of prematurity. The obstetric risk increases the chances of newborns requiring PPV in the delivery room, as found in the present study. However, late preterm birth significantly increases the necessity for PPV. Therefore, considering the morbidity for neonates born between 34 and 37 weeks of gestational age, efforts should be focused on minimizing the unnecessary premature birthrate and improving the outcome of these children [16, 17].

In our study, C-section in labor was associated with the need for PPV and oxygen in the delivery room. However, after adjustments for maternal disorders and gestational age, the type of delivery for these outcomes was no longer significant. This finding is different from a previous study in Brazil, where non-urgent cesarean delivery contributed to an increased need for PPV in the delivery room [5]. However, the population of these studies was different. De Almeida et al.’s study only included newborns at term and studied only non-urgent or elective cesarean. Our study included late preterm and C-sections that were performed during labor.

Throughout the world, the most common causes of neonatal death are preterm birth complications, intrapartum-related complications (birth asphyxia), and neonatal sepsis [18]. In the current study, those births that were late preterm were more likely to be subjected to resuscitation in the delivery room. Therefore, actions that could avoid these circumstances are key to reducing risks. Anticipation, adequate preparation, accurate evaluation, and prompt initiation of support are critical steps for successful neonatal resuscitation. Knowledge about risk factors in the delivery room is essential for avoiding severe birth asphyxia and death as well as helping to plan adequate care.

## Conclusions

Late preterm infants, previous maternal obstetric risks and extremes of maternal age contributed to the higher needs of PPV and use of supplementary oxygen O2 in the delivery room. These characteristics need to be considered in planning care in the delivery room. The risk anticipation can help the team to do adjustments for improvement in quality of care in delivery room.
